# Climate variability in the northern and southern Altai Mountains during the past 50 years

**DOI:** 10.1038/s41598-018-21637-x

**Published:** 2018-02-19

**Authors:** Dongliang Zhang, Yunpeng Yang, Bo Lan

**Affiliations:** 1 Xinjiang Institute of Ecology and Geography, Chinese Academy of Sciences, 818 Beijing South Road, Urumqi, 830011 China; 2 University of Chinese Academy of Sciences, 19A Yuquan Road, Beijing, 100049 China; 3 School of Environmental and Chemical Engineering, Chongqing Three Gorges University, Chongqing, 404000 China

## Abstract

The Holocene drying trend in the northern Altai Mountains and the wetting trend in the southern Altai Mountains inferred from the paleoclimatic studies indicated it is needed to understand the modern climatic characters in this region. However, a detailed analysis of modern climate variations in the northern and southern Altai Mountains is lacking. Here, we investigate the monthly temperature and monthly precipitation data from seventeen meteorological stations during 1966–2015 in the northern and southern Altai. The result shows that temperature increases significantly in the northern (0.42 °C/10 yr) and in the southern (0.54 °C/10 yr). The precipitation decreases insignificantly (−1.41 mm/10 yr) in the northern, whereas it increases significantly (8.89 mm/10 yr) in the southern. The out-of-phase relationship of precipitation changes is also recorded at different time-scales (i.e., season, year, multi-decades, centennial and millennial scales), indicating the Altai Mountains are an important climatic boundary. Based on the analysis of modern atmosphere circulation, the decreased precipitation in the northern corresponds to the decreasing contribution of ‘Northern meridional and Stationary anticyclone’ and ‘Northern meridional and East zonal’ circulation and the increased precipitation in the southern are associated with the increasing contribution of ‘West zonal and Southern meridional’ circulation.

## Introduction

**Figure 1:**
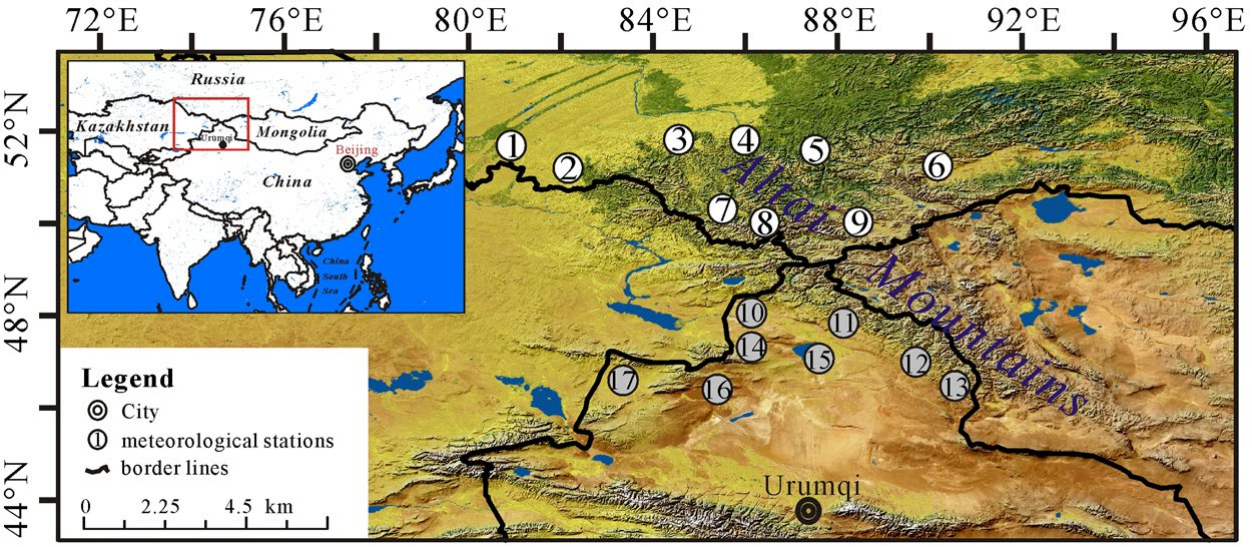
Geographic location of the Altai Mountains and the mentioned meteorological stations (1-Rubcovsk; 2-Zmeinogorsk; 3-Soloneshnoe; 4-Kyzyl-Ozek; 5-Yailu; 6-Mugur-Aksy; 7-Ust-Coksa; 8-Kara-Tyurek; 9-Kosh-Agach; 10-Habahe; 11-Aletai; 12-Fuyun; 13-Qinghe; 14-Jeminay; 15-Fuhai; 16-Hoboksar and 17-Tacheng). Notes: the map is created via the software ArcGIS 10.1 and the related research marks and words are added using the CorelDRAW Graphics Suite 12. The data of the map is from the site: http://due.esrin.esa.int/page_globcover.php.

The Altai Mountains, one of the most prominent mountain ranges in Central Asia (Fig. 1), are important both ecologically and climatologically^1,2^. It is not only an important ecological transition where the Taiga forests in the north have interacted with the steppes in the south and also an important climatic conjunction where the North Atlantic climate systems from the west have interacted with the Pacific climate systems from the east^1,2^. The abundant studies about the modern climate change in northern Xinjiang including the southern Altai Mountains has revealed the rising trend of the past 50-year precipitation under a consistently warming condition^1–14^. However, no more attention is detailed paid on the different geographic units (e.g., the northern slope and the southern slope) of the mountains (i.e., the Tianshan Mountains and the Altai Mountains). Interestingly, the paleoclimatic studies showed that spatially and temporally different Holocene vegetation and climate histories in the different geographical regions of the Altai Mountains^15–19^. In detail, in the northern Altai within Russia the early Holocene (~10,000–~5000 cal. yr BP) was warm and wet and the late Holocene (~5000–0 cal. yr BP) was cold and dry. In the southern Altai within China the climate exhibited a warm-dry early Holocene and a cold-wet late Holocene. To understand the characters of modern climate in the different geographical units of the Altai Mountains, we investigate the monthly temperature and precipitation data from seventeen meteorological stations during 1966–2015 in the northern and southern Altai Mountains. It is helpful to provide a climatic background for protection of ecology and water resources of the Altai Mountains and also contribute to our understanding about the past climatic changes (e.g. Holocene) in the different geographical units of the Altai Mountains.

## Study Area

The Altai Mountains, situated in the middle part of Europe-Asia continent, stretch over Russia, Mongolia, Kazakhstan and China. It covers an extension of more than 1200 km along northwest-southeast direction (Fig. 1). According to the meteorologically documented data, the westerly airflow prevails over the Altai Mountains throughout a year and the Siberian High dominates the Altai Mountains during winter^1,20^. The precipitation in the Altai Mountains is characterized by a decreasing eastward trend and more precipitation on western and northern sides of the mountains^1^. For example, the precipitation in Kanas Lake in the western part reaches up to about 1000 mm, while that in Qinghe in the eastern part is less than 200 mm^2^. Due to the topography effect, the precipitation increases with altitude: ~200–~300 mm in the low-mountain belt, ~300–~500 mm in the middle-mountain belt and above 600 mm in the high-mountain belt^1,2^. In addition, the Altai Mountains contain abundant water resources and gestate great water systems including the Irtysh River, the Wulungu River, the Katun River and the Biya River.

The selected locations of seventeen meteorological stations are shown in Fig. 1 and detailed information is shown in Table 1. The nine meteorological stations of the northern Altai Mountains within Russia are Rubcovsk, Zmeinogorsk, Soloneshnoe, Kyzyl-Ozek, Yailu, Mugur-Aksy, Ust-Coksa, Kara-Tyurek and Kosh-Agach. These meteorological data from Russia sets have been automatically processed for quality as well as homogeneity control before being stored at the RIHMI-WDC. The RIHMI is the major source of official information of the Russian meteorological stations. The eight meteorological stations of the southern Altai Mountains within China are Habahe, Aletai, Fuyun, Qinghe, Jeminay, Fuhai, Hoboksar and Tacheng. These data from China also have been pre-disposed through the strict quality control and homogenized by China Meteorological Administration. The analytical interval is 1966–2015. Two notes should be pointed out. Firstly, no open meteorological data have been published in the eastern part of the Altai Mountains within Mongolia. Secondly, the observed interval (e.g., Katongtuolegai, 49.17°N, 85.62°E, 1072 m a.s.l.) in the western part of the Altai Mountains within Kazakhstan just extends to 2006 and no data are recorded after 2006.

**Table 1:** The related information of meteorological stations in the northern and southern Altai Mountains.

Location	No.	Station Name	Latitude (°N)	Longitude (°E)	Altitude (m)	Annual Temperature (□)	Annual Precipitation (mm)
Northern Altai Mountains	1	Rubcovsk	51.58	81.20	216	2.83	341.05
	2	Zmeinogorsk	51.15	82.17	354	2.81	692.78
	3	Soloneshnoe	51.63	84.33	409	1.86	581.20
	4	Kyzyl-Ozek	51.90	86.00	331	2.07	745.00
	5	Yailu	51.77	87.60	480	3.73	894.95
	6	Mugur-Aksy	50.38	90.43	1850	−2.36	142.60
	7	Ust-Coksa	50.30	85.60	978	0.62	472.23
	8	Kara-Tyurek	50.00	86.40	2600	−5.54	601.41
	9	Kosh-Agach	50.00	88.04	1760	−4.96	119.93
Southern Altai Mountains	10	Habahe	48.05	86.40	534	4.77	199.93
	11	Aletai	47.03	88.08	736.9	4.48	190.13
	12	Fuyun	46.98	85.52	826.6	2.88	177.71
	13	Qinghe	46.67	90.38	1220	0.72	125.01
	14	Jeminay	47.43	85.87	984	4.29	215.07
	15	Fuhai	46.98	89.52	496	4.18	191.87
	16	Hoboksar	46.78	85.72	1314	3.87	142.43
	17	Tacheng	46.73	83.00	540	7.24	293.28

## Results

### Change in temperature

#### Temperature variations in the northern Altai Mountains

**Figure 2:**
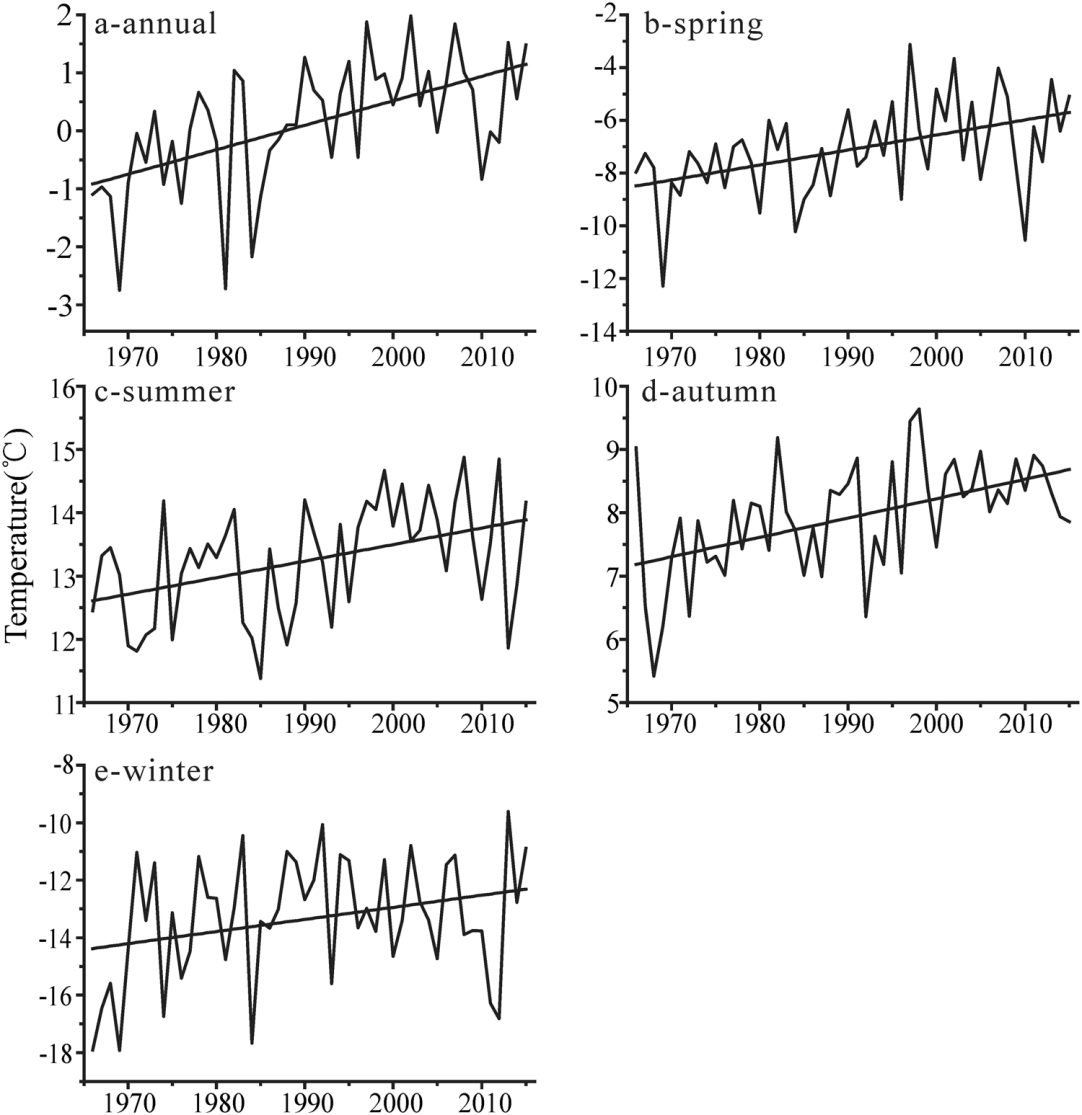
Mean changes in the temperature of the northern Altai Mountains: annual (**a**), spring (**b**), summer (**c**), autumn (**d**) and winter (**e**).

The Mann-Kendall (MK) statistical test showed that there is a significant increasing trend in the mean annual temperature of the northern Altai Mountains during the 1966–2015 time frame (P < 0.01) (Fig. 2a, Table 2). Ust-Coksa, among the stations in the northern Altai Mountains has the largest temperature tendency (i.e., increasing rate, 0.47 °C/10 yr), and Kara-Tyurek has the lowest climate tendency (i.e., increasing rate, 0.32 °C/10 yr). Overall, temperature significantly increases with a rate of 0.42 °C/10 yr (P < 0.01) in the northern Altai Mountains (Fig. 2a).

**Table 2:** The annual and seasonal mean temperatures and the corresponding climate tendencies in the northern Altai Mountains during 1966–2015.

	Station Name	Annual	Spring	Summer	Autumn	Winter
Mean Temperature (□)	Rubcovsk	2.76	−5.95	17.61	11.45	−11.75
	Zmeinogorsk	2.81	−5.38	16.43	10.71	−10.60
	Soloneshnoe	1.66	0	15.41	9.81	−12.15
	Kyzyl-Ozek	2.47	−5.06	15.69	10.14	−10.85
	Yailu	3.93	−2.19	13.75	10.02	−5.62
	Mugur-Aksy	−2.15	−9.29	11.05	5.49	−16.72
	Ust-Coksa	−0.35	−6.95	13.63	8.07	−15.80
	Kara-Tyurek	−5.36	−11.4	4.01	0.21	−14.17
	Kosh-Agach	−4.34	−11.86	11.51	5.41	−22.43
Climate Tendency (□/10 yr)	Rubcovsk	0.42**	0.51**	0.21*	0.27*	0.55**
	Zmeinogorsk	0.38**	0.60**	0.14*	0.31**	0.30*
	Soloneshnoe	0.40*	0.69**	0.11*	0.21*	0.47**
	Kyzyl-Ozek	0.46**	0.72**	0.20*	0.33**	0.58*
	Yailu	0.38**	0.52**	0.26**	0.31*	0.33**
	Mugur-Aksy	0.44**	0.43**	0.54**	0.33**	0.25*
	Ust-Coksa	0.47**	0.62**	0.19*	0.22*	0.69**
	Kara-Tyurek	0.32**	0.49**	0.30**	0.39**	0.18
	Kosh-Agach	0.42**	0.56**	0.33**	0.35**	0.44*
	Average	0.42**	0.68**	0.26**	0.31**	0.42**

Note: * and ** indicate that the climate tendency is significant at the level of 0.05 and 0.01, respectively, by the Mann-Kendall test for the long-term trend.

In terms of the seasonal consistencies or inconsistencies of the changes in temperature, increasing trends are statistically detectable for all seasons among nine stations of the northern Altai Mountains (Fig. 2, Table 2). Spring (Fig. 2b) is the season when the temperature increases most dramatically with a rate of 0.68 °C/10 yr (P < 0.01), and the second increased season is winter (0.42 °C/10 yr, P < 0.01). The fastest increasing temperature of spring is observed in Kyzyl-Ozek (0.72 °C/10 yr, P < 0.01) and the lowest is in Mugur-Aksy (0.43 °C/10 yr, P < 0.01). The increasing rate of winter temperature at Ust-Coksa is much lower (0.69 °C/10 yr, P < 0.01) and no significant increase of temperature is observed in winter in Kara-Tyurek (0.18 °C/10 yr). The temperature in summer and autumn is also increased significantly with average rates of 0.26 °C/10 yr and 0.31 °C/10 yr, respectively (Fig. 2c,d).

In addition, the obvious altitudinal differences of seasonal temperature are showed among nine stations in the northern Altai Mountains though no obvious differences in annual changes. Specifically, the increased rates of spring and winter in high-altitude stations (i.e., Mugur-Aksy, Kara-Tyurek and Kosh-Agach) are lower than that in low-altitude stations (i.e., Rubcovsk, Zmeinogorsk, Soloneshnoe, Kyzyl-Ozek, Yailu and Ust-Coksa), whereas the increased rates of summer and autumn of high-altitude stations are higher than that of low-altitude stations.

#### Temperature variations in the southern Altai Mountains

**Figure 3:**
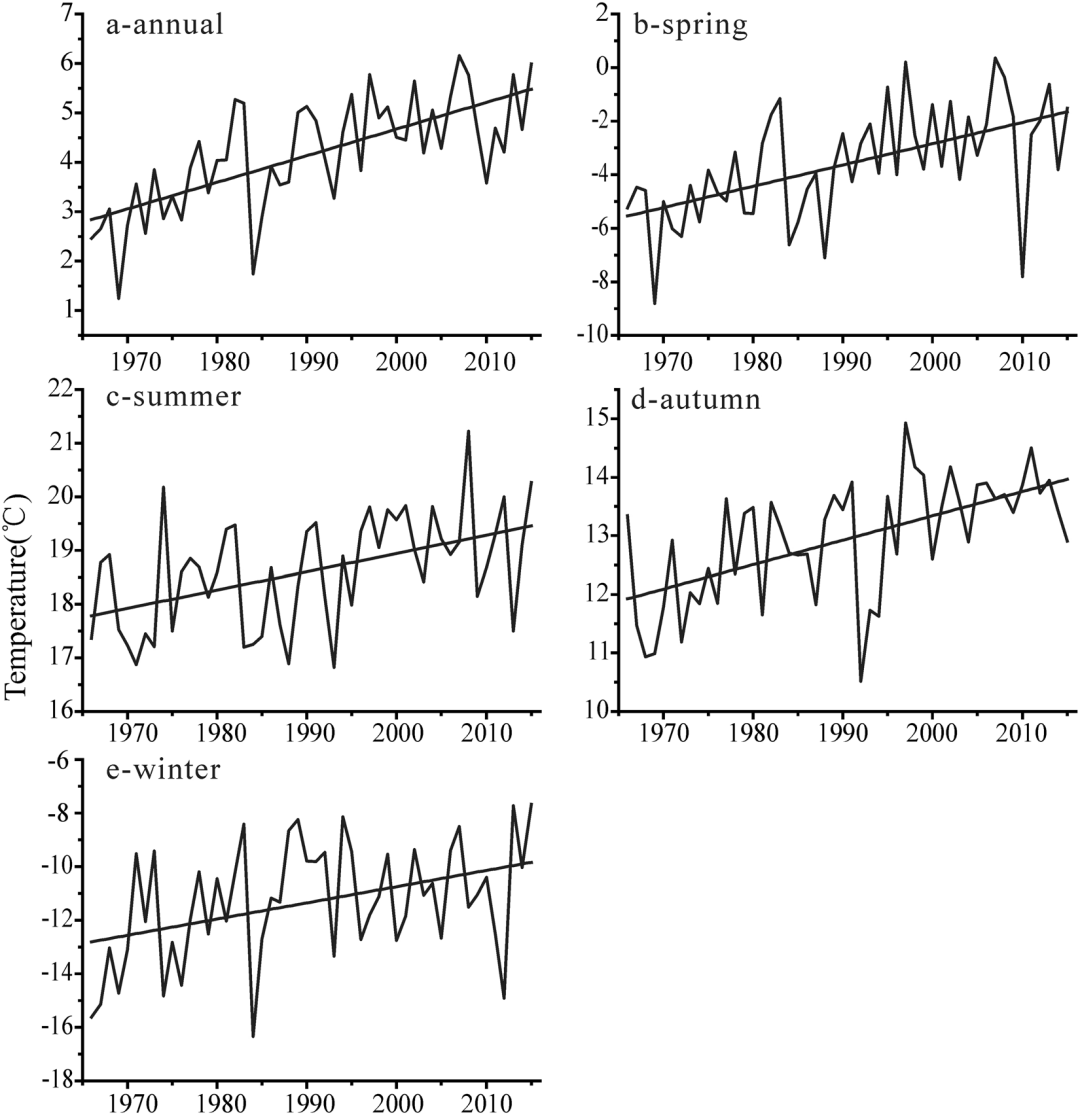
Mean changes in the temperature of the southern Altai Mountains: annual (**a**), spring (**b**), summer (**c**), autumn (**d**) and winter (**e**).

Consistent with the increasing temperature in the northern Altai Mountains, the mean annual temperature also increases significantly (P < 0.01) with a rate of 0.54 °C/10 yr in the southern Altai Mountains over the past 50 years (Fig. 3, Table 3). Fuyun among the stations has the highest temperature tendency (i.e., increasing rate, 0.81 °C/10 yr), and Aletai has the lowest climate tendency (i.e., increasing rate, 0.33 °C/10 yr).

**Table 3:** The annual and seasonal mean temperatures and the corresponding climate tendencies in the southern Altai Mountains during 1966–2015.

	Station Name	Annual	Spring	Summer	Autumn	Winter
Mean Temperature (□)	Habahe	4.96	−3.19	19.33	13.74	−10.04
	Aletai	4.51	−3.61	19.25	13.53	−11.13
	Fuyun	3.19	−5.22	19.25	13.24	−14.50
	Qinghe	8.74	−6.69	16.51	10.58	−10.90
	Jeminay	4.29	−3.35	17.53	12.21	−9.22
	Fuhai	4.35	−4.15	20.48	13.91	−12.84
	Hoboksar	3.87	−2.76	16.52	11.16	−9.45
	Tacheng	7.24	0.20	20.09	15.18	−6.49
Climate Tendency (□/10 yr)	Habahe	0.45**	0.68**	0.25**	0.36**	0.51*
	Aletai	0.33**	0.69**	0.09	0.11*	0.44**
	Fuyun	0.81**	1.15**	0.51**	0.57**	1.02**
	Qinghe	0.67**	0.94**	0.45**	0.47**	0.81**
	Jeminay	0.49**	0.74**	0.33**	0.47**	0.41**
	Fuhai	0.55**	0.68**	0.38**	0.48**	0.65**
	Hoboksar	0.41**	0.59**	0.28*	0.39**	0.38**
	Tacheng	0.61**	0.89**	0.42**	0.48**	0.62**
	**Average**	**0**.54**	**0**.79**	**0**.34**	**0**.42**	**0**.61**

Note: * and ** indicate that the climate tendency is significant at the level of 0.05 and 0.01, respectively, by the Mann-Kendall test for the long-term trend.

The seasonal pattern in the southern Altai Mountains increases most dramatically during spring with a rate of 0.79 °C/10 yr (P < 0.01) and then winter with a rate of 0.61 °C/10 yr (P < 0.01), being consistent with seasonal changes in the northern Altai Mountains but being larger than the latter. This feature suggests that temperature increases more pronouncedly in the cold season. In addition, the temperature in summer and autumn increases significantly with average rates of 0.34 °C/10 yr and 0.42 °C/10 yr, respectively (Fig. 3c,d). It should be noted that we don’t consider the attitudinal gratitude of temperature in the southern Altai Mountains because of all stations located in the low-altitude region (below 1500 m a.s.l.).

### Change in precipitation

#### Precipitation variations in the northern Altai Mountains

**Figure 4:**
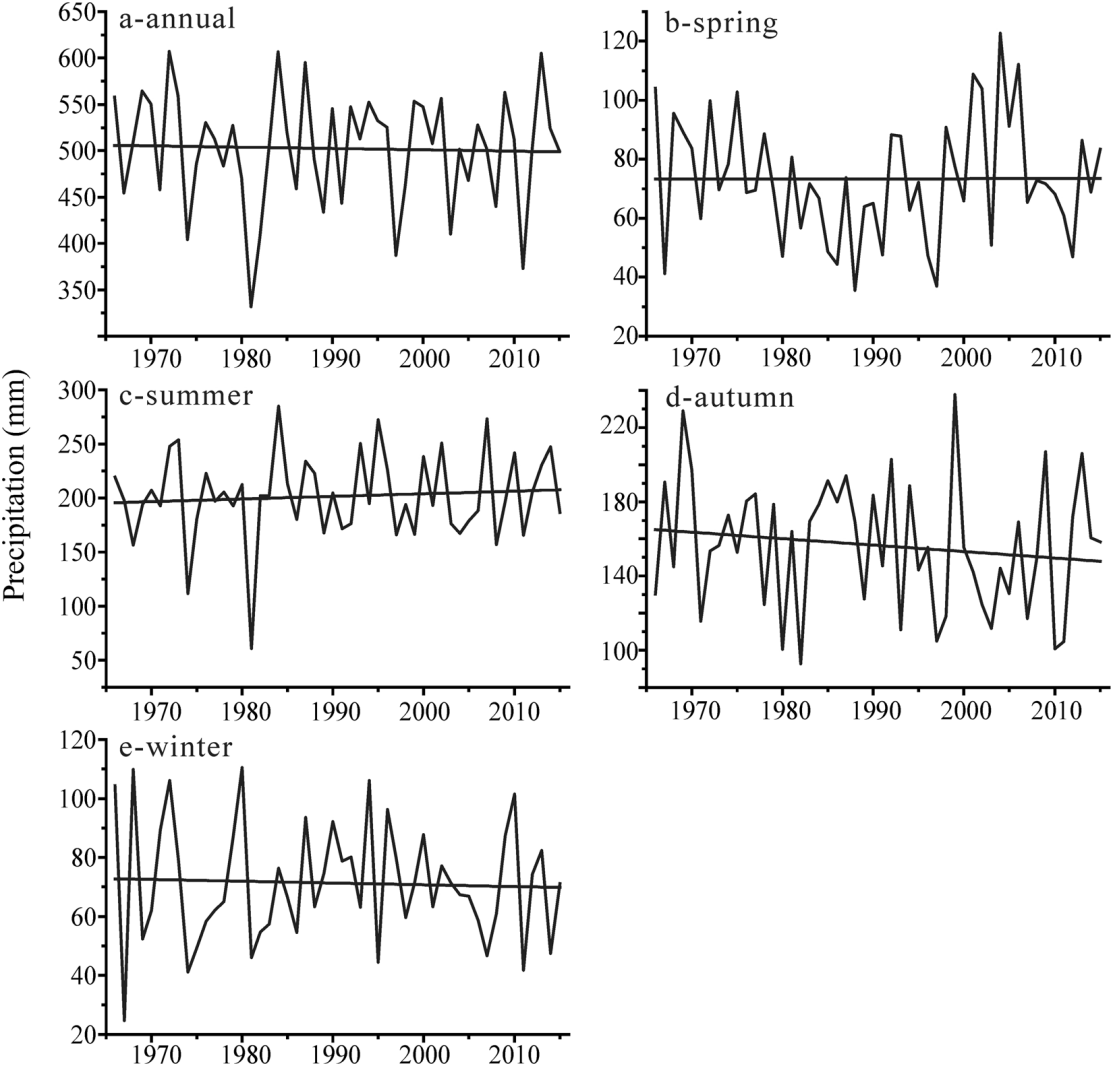
Mean changes in the precipitation of the northern Altai Mountains: annual (**a**), spring (**b**), summer (**c**), autumn (**d**) and winter (**e**).

With the temperature increase, the mean annual precipitation shows various tendencies in the northern Altai Mountains (Fig. 4a, Table 4). In detail, the mean annual precipitation experiences a significant increasing trend in Soloneshnoe with a rate being 12.37 mm/10 yr and that increases insignificantly in Yailu (3.77 mm/10 yr), Ust-Coksa (2.19 mm/10 yr) and Kosh-Agach (0.53 mm/10 yr). Conversely, the mean annual precipitation decreases significantly in Rubcovsk (−5.20 mm/10 yr, P < 0.05), Zmeinogorsk (−6.53 mm/10 yr, P < 0.05), Kyzyl-Ozek (−10.08 mm/10 yr, P < 0.01), Mugur-Aksy (−12.32 mm/10 yr, P < 0.01) and Kara-Tyurek (−2.75 mm/10 yr). Overall, mean annual precipitation in the northern Altai Mountains decreases with −1.41 mm/10 yr.

**Table 4:** The annual and seasonal mean precipitation and the corresponding climate tendencies in the northern Altai Mountains during 1966–2015.

	Station Name	Annual	Spring	Summer	Autumn	Winter
Mean Precipitation (mm)	Rubcovsk	336.70	56.15	127.79	91.26	61.49
	Zmeinogorsk	689.17	137.06	201.96	178.28	171.87
	Soloneshnoe	572.02	88.09	242.85	170.09	70.99
	Kyzyl-Ozek	733.56	111.38	282.94	238.22	101.03
	Yailu	879.96	117.44	374.85	299.44	88.22
	Mugur-Aksy	139.32	9.23	72.22	42.61	15.25
	Ust-Coksa	470.19	51.37	208.05	155.10	55.68
	Kara-Tyurek	574.46	81.63	235.77	191.71	65.36
	Kosh-Agach	118.35	7.36	61.81	37.23	11.96
Climate Tendency (mm/10 yr)	Rubcovsk	−5.20*	−4.15	2.24	−1.23	2.05
	Zmeinogorsk	−6.53*	−3.72	4.67	−7.22*	0.25
	Soloneshnoe	12.37**	2.81	11.88**	3.29	0.77
	Kyzyl-Ozek	−10.08**	−1.29	2.78	−11.66**	−2.49
	Yailu	3.77	3.72	6.13*	−6.47*	0.39
	Mugur-Aksy	−12.32**	0.22	−6.85*	−4.23	−1.02
	Ust-Coksa	2.19	1.15	−0.51	−0.12	1.68
	Kara-Tyurek	−2.75	−0.06	0.11	−1.11	−1.70
	Kosh-Agach	0.53	−0.34	1.57	−0.28	−0.43
	**Average**	**−1**.**41**	**0**.**04**	**2**.**45**	**−3**.**47**	**−0**.**60**

Note: * and ** indicate that the climate tendency is significant at the level of 0.05 and 0.01, respectively, by the Mann-Kendall test for the long-term trend.

In terms of the seasonal consistencies or inconsistencies of the changes in precipitation, various trends are also statistically detectable for all seasons during the past 50 years (Fig. 4b–e). Specifically, precipitation in spring has no obvious changes and that in summer experiences a slightly and insignificantly increased rate of 2.45 mm/10 yr. Interestingly, precipitation in autumn among almost all stations shows a decreasing trend with a maximum decreased rate (−11.66 mm/10 yr, P < 0.01) in Kyzyl-Ozek although precipitation insignificantly decreases in other stations.

#### Precipitation variations in the southern Altai Mountains

**Figure 5:**
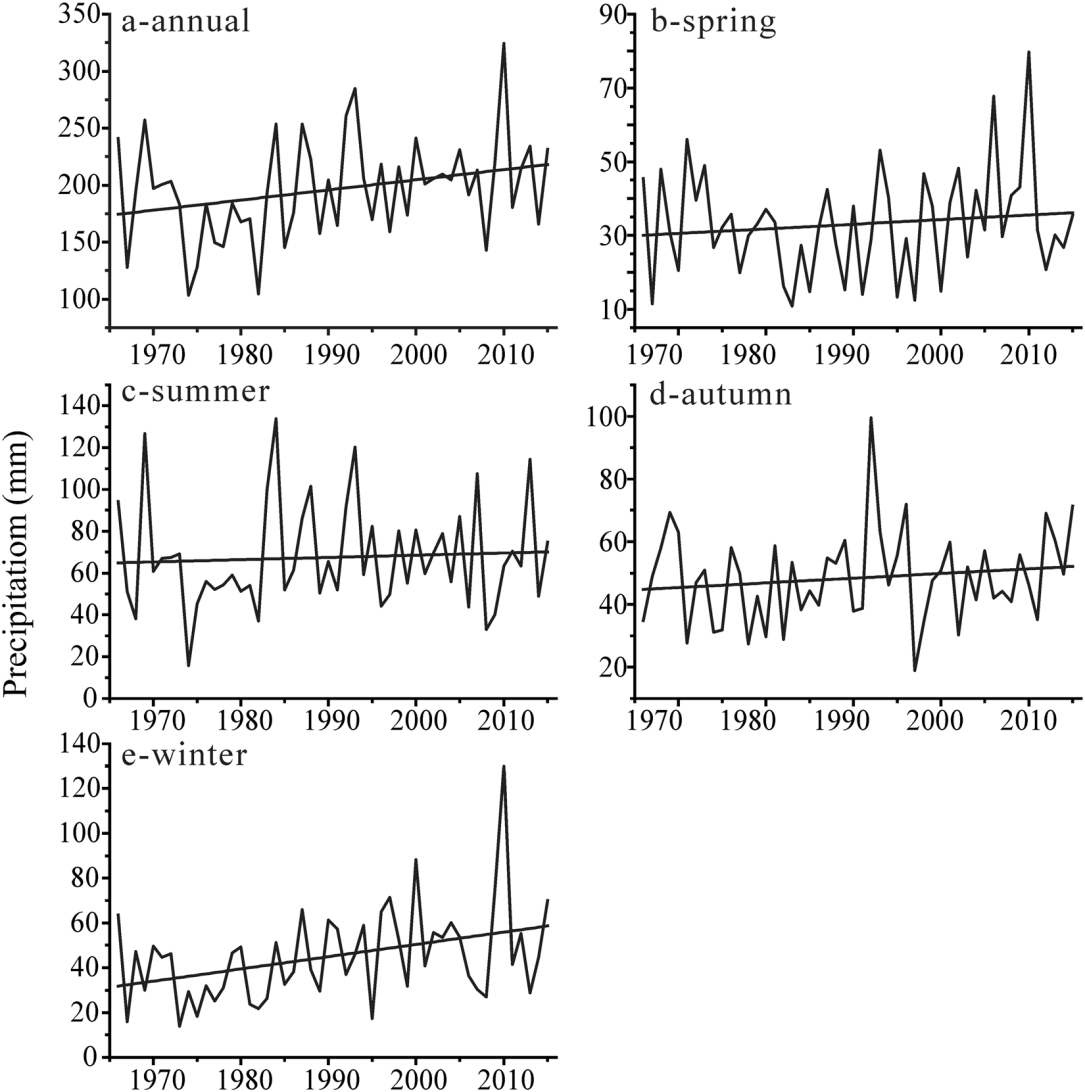
Mean changes in the precipitation of the southern Altai Mountains: annual (**a**), spring (**b**), summer (**c**), autumn (**d**) and winter (**e**).

Compared with the varied trends of precipitation in the northern Altai Mountains, the precipitation significantly (P < 0.01) has kept a consistent rising trend with a rate 8.89 mm/10 yr in the southern Altai Mountains (Fig. 5a, Table 5), being similar with the average rate (about 8.40 mm/10 yr) in Central Asia for the same period^3^. The maximum increased rate of precipitation in Habahe is 14.71 mm/10 yr, the minimum rate in Tacheng is only 2.27 mm/10 yr.

**Table 5:** The annual and seasonal mean precipitation and the corresponding climate tendencies in the southern Altai Mountains during 1966–2015.

	Station Name	Annual	Spring	Summer	Autumn	Winter
Mean Precipitation (mm)	Habahe	197.53	36.76	64.23	51.73	44.80
	Aletai	203.37	34.39	58.92	49.66	60.41
	Fuyun	197.03	32.61	65.11	47.11	52.21
	Qinghe	180.07	25.94	65.95	46.44	41.74
	Jeminay	215.07	38.70	75.58	54.76	46.02
	Fuhai	125.70	15.94	48.12	39.76	21.87
	Hoboksar	142.43	15.79	75.66	38.25	12.72
	Tacheng	293.28	64.80	86.62	59.82	82.04
Climate Tendency (mm/10 yr)	Habahe	14.71**	4.42**	−0.88	2.79*	8.38**
	Aletai	12.28**	1.36	1.37	2.21	7.35**
	Fuyun	12.45**	1.14	4.15*	1.04	6.13**
	Qinghe	9.39**	1.25	3.91*	0.31	3.91*
	Jeminay	11.54**	1.28	0.31	2.63	7.32**
	Fuhai	5.85**	0.99	0.86	0.03	3.96**
	Hoboksar	5.77**	1.18	1.62	1.06	1.91
	Tacheng	2.27	−1.62	−2.83	1.87	4.84**
	**Average**	**8**.89**	**1**.**25**	**1**.**06**	**1**.**49**	**5**.47**

Note: * and ** indicate that the climate tendency is significant at the level of 0.05 and 0.01, respectively, by the Mann-Kendall test for the long-term trend.

For precipitation in all seasons, increasing trends are statistically detectable for all stations during the past ~50 years (Fig. 5b–e). However, the Manne-Kendall trend test showed that the increase precipitation in winter is significant, while the increase in spring, summer and autumn is insignificant. The Manne-Kendall trend test also showed that the precipitation in spring significantly increases in Habahe, while that in summer significantly increases in Fuyun and Qinghe. It is worth noting that winter is also the season when the precipitation increased most dramatically among four seasons. The fastest increasing winter precipitation is observed in Habahe (8.38 mm/10 yr) and the lowest in Hoboksar (1.91 mm/10 yr). In general, an increase of precipitation is the main character of precipitation in the southern Altai Mountains.

## Comparisons and Discussions

Based on the above-mentioned analysis, we can find that the temperature experiences an obviously increased trend by a rate of 0.42 °C/10 yr in the northern Altai Mountains and by a rate of 0.54 °C/10 yr in the southern Altai Mountains during 1966–2015. The increased temperature rate in the southern is larger than that in the northern, which might be attributed to higher annual precipitation amounts in the northern. The markedly increased temperature rates in the northern and southern Altai are both larger than the average of northwest China (0.34 °C/10 yr)^4–6^, the whole China (0.25 °C/10 yr)^7^ and the entire globe (0.175 °C/10 yr)^8^. In terms of the seasonal changes in temperature of the northern and southern Altai, the temperature increases most dramatically in the cold season (spring and winter).

Under the warming condition, the mean annual precipitation decreases insignificantly by −1.41 mm/10 yr in the northern Altai during 1966–2015, consistent with the results in the eastern Altai Mountains^9^. Though no consistently changeable trends in spring, summer and winter, the changes of precipitation in autumn consistently reduce. Conversely, the mean annual precipitation increased significantly by a rate of 8.89 mm/10 yr in the southern Altai Mountains, being similar with the changeable trend of precipitation in northwest China (including north Xinjiang)^3–5,10–12^ and Central Asia^13,14^. In terms of seasonal changes, winter is the season when the precipitation increased most dramatically. Overall, the decrease of precipitation in the northern Altai during 1966–2015 is attributed to the decrease of autumn precipitation, while the increase of precipitation in the southern Altai could result from the increase of winter precipitation.

**Figure 6:**
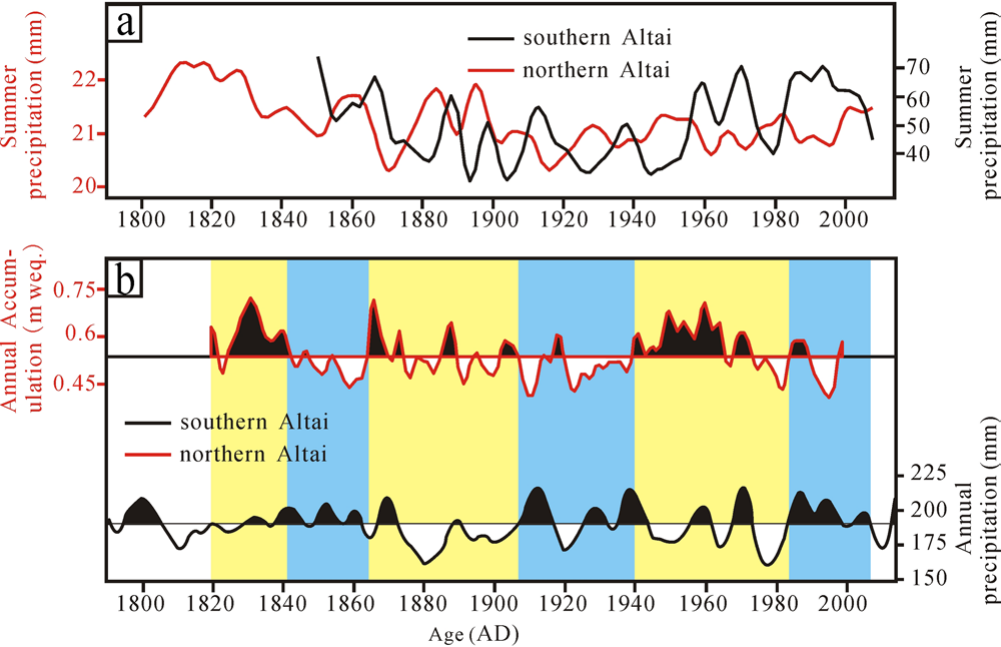
Comparison of summer (**a**) and annual (**b**) precipitation variations during the past 200 years in the northern and southern Altai Mountains

According to the changeable trends of temperature and precipitation during the past 50 years (1966–2015), the climate experiences a drying trend in the northern Altai, whereas the climate exhibits a wetting trend in the southern Altai. We therefore propose that the climate changes are out-of-phase between the northern Altai and the southern Altai. This proposal is supported by tree-ring- and ice core-recorded (Fig. 1) precipitation variations in the past two hundred years^21–24^. Specifically, Fig. 5a presents two tree-ring oxygen isotope-indicated summer precipitation amount curves of the past two hundred years in the northern^21^ and southern Altai Mountains^22^. The result reveals that changes of summer precipitation amounts in the northern (red curve in Fig. 6a) and in the southern (dark curve in Fig. 6a) are totally opposite. The opposite trend is also recorded in annual precipitation variations in the northern (red curve in Fig. 6b) and in the southern (dark curve in Fig. 6b) during the past two hundred years^23,24^. It should be noted that summer and annual precipitation both have a decreasing trend in the northern Altai and experience an increasing trend in the southern Altai, providing a strong support to the aforementioned proposition that the climate change is out-of-phase between the northern Altai and the southern Altai.

Furthermore, the out-of-phase relationship of precipitation in the northern and southern Altai Mountains also exists in the last millennium^25,26^ and the Holocene epoch^15–19^. In detail, the climate is characterized by a relatively wet condition in the Medieval Warm Period (800–1200 AD) and a relatively dry condition in the Little Ice Age (1400–1800 AD) in the northern Altai Mountains^25^, while the climate by a relatively dry condition in the Medieval Warm Period and a relatively wet condition in the Little Ice Age in the southern Altai Mountains^26^. During the Holocene epoch, the climate is featured by a wet condition in the early Holocene warm period (between ~10,000 and ~5000 cal. yr BP) and that by a dry condition in the late Holocene cold period (between ~5000 and 0 cal. yr BP) in the northern Altai Mountains and the totally converse Holocene climatic condition has been showed in the southern^15–19^. Therefore, we can conclude that the out-of-phase relationship of precipitation change at different time-scales (i.e., season, year, multi-decades, centennial and millennial scales) indicates that the Altai Mountains are an important climatic boundary. The vegetation evolution^2^ and oxygen isotopes of precipitation^27^ strongly support this result. Specifically, the northern Altai Mountains are dominated by the densely covered forests and the relatively depleted oxygen isotope of precipitation (averaged −12‰), whereas the southern are dominated by the Asian cold steppe and the relatively enriched oxygen isotope of precipitation (averaged −7‰).

**Figure 7:**
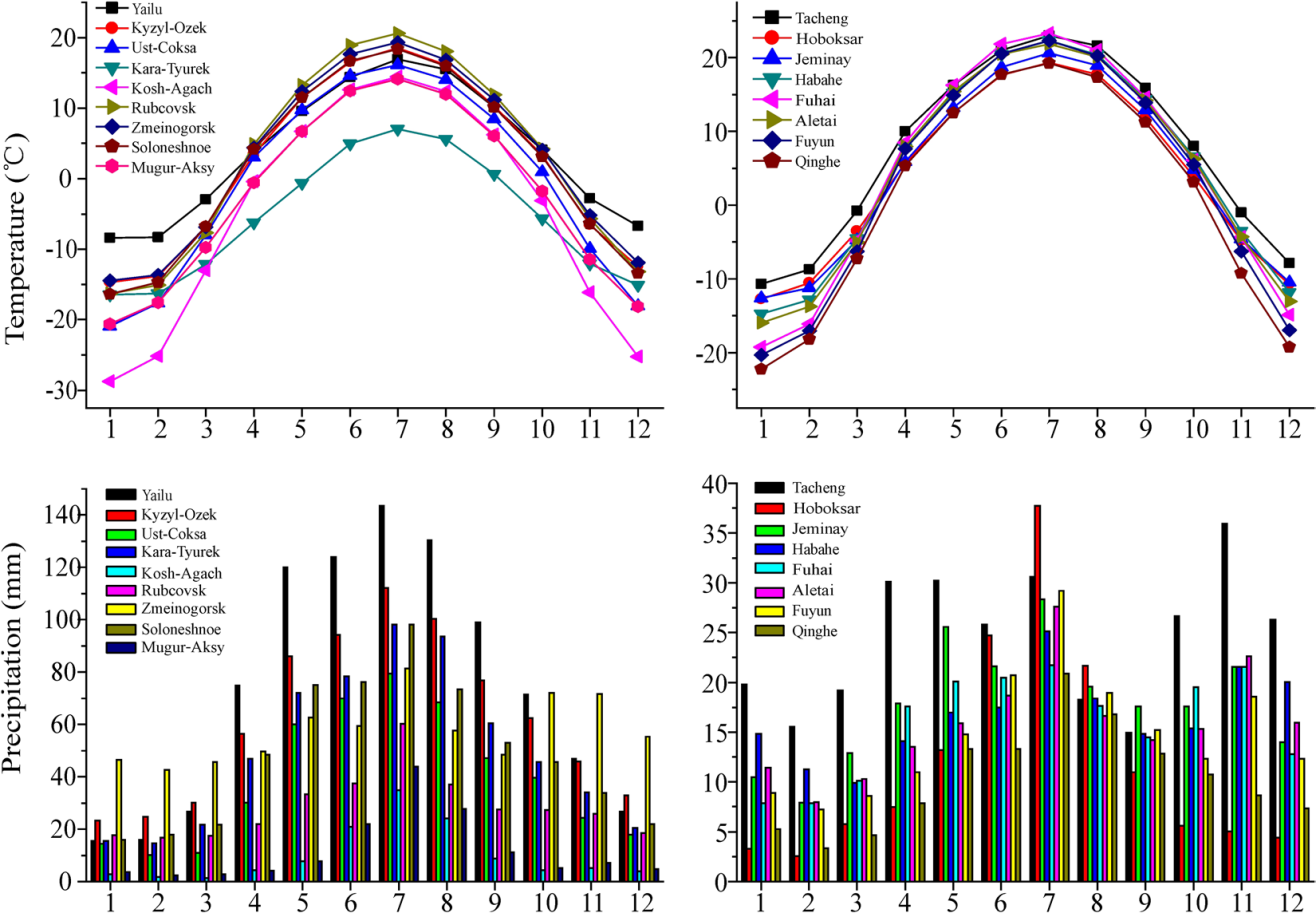
Average monthly temperature and precipitation change in the northern Altai Mountains (left) and the southern Altai Mountains (right) during 1966–2015.

Under the large-scale wetting condition during 1966–2015 in Central Asia (including the Altai Mountains)^1–14,28–33^, the reason of diverging (i.e., opposite) trend of precipitation in the northern and southern Altai Mountains over the past 50 years should be taken into consideration. Our attention firstly turns to the seasonal characters of precipitation in the northern and southern Altai (Fig. 7). The unimodal distribution of precipitation in the northern Altai mostly concentrates in summer and autumn with 55–84%. The largest percentage in Kosh-Agach is about 83.89%. The bimodal distribution of precipitation in the southern Altai Mountains is featured by two maximum in April-September (50–68%) and in November-December (13–21%). The largest percentage of April-September precipitation is about 68.05% in Fuhai, and the largest percentage of November-December is about 21.22% in Tacheng^34^.

Our attention in turn changes to the resource of water vapor in the northern and southern Altai Mountains. Three supply channels of water vapor shape regional climate of the Altai Mountains and they are (1) the eastward water vapor flow transferred by western and northwestern intrusions from the Atlantic Ocean; (2) the northwestward water vapor by southwest cyclones from the Mediterranean Sea, Black Sea, Caspian Sea and Aral Sea; (3) the southward water vapor flow from the Arctic Ocean^2,32^. The first (55%) and second (34%) water vapor supply channels are mainly responsible for annual precipitation (about 87%) in the northern Altai Mountains, whereas the second (86%) water vapor supply for the southern Altai Mountains^15^. Malygina *et al*.^9^ estimated the influence of atmospheric circulations on these changes and found that the decreased precipitation changes in the northern Altai Mountains correspond to the decreasing contribution (about 3–5%) of ‘Northern meridional and Stationary anticyclone’ and ‘Northern meridional and East zonal’ circulation. For the southern Altai Mountains, the increased precipitation changes are associated with more water vapor supplies from Mediterranean Sea, Black Sea, Caspian Sea and Aral Sea under the increasing contribution (about 15%) of ‘West zonal and Southern meridional’ circulation. The instrumental increased precipitation was detected in the northern Tianshan Mountains^12^ and in the northern Pakistan^35^. Tree-ring-recorded high precipitation variability in the past 50 years was also showed in the western Tianshan Mountains^36–40^ and in the southern Kazakhstan^41^.

## Conclusions

The temperature and precipitation variations are investigated from seventeen meteorological stations during 1966–2015 in the northern and southern Altai Mountains. The results show that the temperature experiences a consistently increasing rate in the northern and southern Altai Mountains. The precipitation in the northern Altai insignificantly decreases, whereas that in the southern Altai significantly increases. The out-of-phase relationship of precipitation variations is also recorded at different time-scales (i.e., season, year, multi-decades, centennial and millennial scales), indicating that the Altai Mountains are an important climatic boundary. Our works are not only conducive to understand the past climatic differences in the Altai Mountains and also imply that the protection of ecology and water resources should take the climatic differences in the whole Altai Mountains into account. Additionally, the influence mechanism of differences between north and south in-depth from the scale of atmospheric circulation are needed to further analyse in the different scales (i.e., season, year, multi-decades, centennial and millennial scales).

## Data Source and Methods

In this study, the monthly temperature and precipitation from seventeen meteorological stations are utilized to investigate their variations during 1966–2015 in the northern and southern Altai Mountains. The selected locations of seventeen meteorological stations are shown in Fig. 1 and detailed information are shown in Table 1. The nine meteorological stations of the northern Altai Mountains within Russia are Rubcovsk, Zmeinogorsk, Soloneshnoe, Kyzyl-Ozek, Yailu, Mugur-Aksy, Ust-Coksa, Kara-Tyurek and Kosh-Agach and the data were downloaded from the website (http://meteo.ru/english/data/). The eight meteorological stations of the southern Altai Mountains within China are Habahe, Aletai, Fuyun, Qinghe, Jeminay, Fuhai, Hoboksar and Tacheng and the data were downloaded from China Meteorological Data Sharing Service System (http://cdc.cma.gov.cn). For the selected seventeen meteorological stations in this study, the annual and seasonal temperatures and precipitations are analyzed over the past 50 years (1966–2015).

The widely-used Manne-Kendall method is an effective test to detect the long-term change in time series^42^. The detailed treated method is seeing in Xu *et al*.^12^. In this study, it was applied to detect the long-term trend change of temperature and precipitation of these seventeen meteorological data.

## References

[1] Aizen EM, Aizen VB, Melack JM, Nakamura T, Ohta T (2001). Precipitation and atmospheric circulation patterns at mid-latitudes of Asia. Int. J. Climatol..

[2] Chen, X. Physical Geography of China’s Arid Zones. Beijing: Science Press (in Chinese) (2010).

[3] Li BF, Chen YN, Chen ZS, Xiong H, Lian L (2016). Why does precipitation in northwest China show a significant increasing trend from 1960 to 2010?. Atmos. Res..

[4] Chen YN, Deng HJ, Li BF, Li Z, Xu CC (2014). Abrupt change of temperature and precipitation extremes in the arid region of Northwest China. Quat. Int..

[5] Li BF, Chen YN, Shi X (2012). Why does the temperature rise faster in the arid region of northwest China. J. Geophys. Res..

[6] Zhang Q, Li JF, Chen YD, Chen XH (2011). Observed changes of temperature extremes during 1960-2005 in China: Natural or human induced variations?. Theor. Appl. Climatol..

[7] Ren GY (2005). Changes of surface air temperature in China during 1951-2004. Clim. Environ. Res..

[8] Harris I, Jones P, Osborn T, Lister DH (2014). Updated high-resolution grids of monthly climatic observations: The CRU TS3. 10 Dataset. Int. J. Climatol..

[9] Malygina N, Papina T, Kononova N, Barlyaeva T (2017). Influence of atmospheric circulation on precipitation in Altai Mountains. J. Moun. Sci..

[10] Li BF, Chen YN, Shi X, Chen ZS, Li WH (2013). Temperature and precipitation changes in the diverse environments in the arid region of northwest China. Theor. Appl. Climatol..

[11] Shi, Y. F. An assessment of the issues of climate shift from warm-dry to warm-wet in northwest China. China Meteorological Press, Beijing, pp. 17–25 (2003).

[12] Xu CC, Li JX, Zhao J, Gao ST, Chen YP (2015). Climate variations in northern Xinjiang of China over the past 50 years under global warming. Quat. Int..

[13] Xu LG, Zhou HF, Du L, Yao HJ, Wang HB (2015). Precipitation trends and variability from 1950 to 2000 in arid lands of CentralAsia. J. Arid Land..

[14] Zhang M, Chen Y, Shen Y, Li Y (2017). Changes of precipitation extremes in arid CentralAsia. Quat. Int..

[15] Chen FH (2008). 2008. Holocene moisture evolution in arid central Asia and its out-of-phase relationship with Asian monsoon history. Quat. Sci. Rev..

[16] Chen FH (2016). A persistent Holocene wetting trend in arid central Asia, with wettest conditions in the late Holocene, revealed by multi-proxy analyses of loess-paleosol sequences in Xinjiang, China. Quat. Sci. Rev..

[17] Feng ZD (2017). *Vegetation chan*ges and associated climatic changes in the southern Altai Mountains within China during the Holocene. Holocene.

[18] Wang W, Feng ZD (2013). Holocene moisture evolution across the Mongolian Plateau and its surrounding areas: A synthesis of climatic records. Earth-Sci. Rev..

[19] Ran M, Feng ZD (2013). Holocene moisture variations across China and driving mechanisms: a synthesis of climatic records. Quat. Inter..

[20] Aizen VB (2006). Climatic and atmospheric circulation pattern variability from ice-core isotope/geochemistry records (Altai, Tian Shan and Tibet). Ann. of Glaciol..

[21] Sidorova OV (2013). The application of tree-rings and stable isotopes for reconstructions of climate conditions in the Russian Altai. Clim. Change..

[22] Xu G (2014). Relative humidity reconstruction for northwestern China’s Altay Mountains using tree-ring δ^18^O. Chinese Sci. Bull..

[23] Chen F (2014). Precipitation reconstruction for the southern Altay Mountains (China) from tree rings of *Siberian spruce*, reveals recent wetting trend. Dendrochronologia.

[24] Henderson KA (2006). Temporal variations of accumulation and temperature during the past two centuries from Belukha ice core, Siberian Altai. J. Geophys. Res..

[25] Andreev AA (2007). Environmental changes in the northern Altai during the last millennium documented in Lake Teletskoye pollen record. Quat. Res..

[26] Li, Y. *et al*. Hydroclimatic changes over the past 900 years documented by the sediments of Tiewaike Lake, Altai Mountains, Northwestern China. *Quat*. *Inter*. 10.1016/j.quaint.2016.07.053 (2016).

[27] Liu XK (2015). Variations in the oxygen isotopic composition of precipitation in the Tianshan Mountains region and their significance for the Westerly circulation. J. Geogr. Sci..

[28] Hu, Z. *et al*. Variations and changes of annual precipitation in Central Asia over the last century. *Inter*. *J*. *Climato*. doi:10.1002/joc.4988 (2017).

[29] Huang W, Chen F, Feng S, Chen J, Zhang X (2013). Interannual precipitation variations in the mid-latitude Asia and their association with large-scale atmospheric circulation. Chin. Sci. Bull..

[30] Yang LM, Zhang QY (2008). Effects of the north atlantic oscillation on the summer rainfall anomalies in Xinjiang. Chin. J. Atmosph. Sci..

[31] Kutzbach JE (2014). Potential role of winter rainfall in explaining increased moisture in the Mediterranean and Middle East during periods of maximum orbitally-forced insolation seasonality. Clim. Dyn..

[32] Li J, Wang JX (2003). A modified zonal index and its physical sense. Geophys. Res. Lett..

[33] Zhang DL, Lan B, Yang YP (2017). Comparison of precipitation variations at different time scales in the northern and southern Altai Mountains. Acta Geographica Sinica.

[34] Li, J. F. Climates of Xinjiang. Beijing: Meteorological Press, 97–124 (in Chinese) (1991).

[35] Treydte KS (2006). The twentieth century was the wettest period in northern Pakistan over the past millennium. Nature.

[36] Zhang RB (2016). *Tree-ring-based moisture variability in wes*tern Tianshan Mountains since A.D. 1882 and its possible driving mechanism. Agricultural and Forest Meteorology.

[37] Zhang RB (2017). A 189-year tree-ring record of drought for the Dzungarian Alatau, arid CentralAsia. J. Asian Earth Sciences.

[38] Chen F (2013). A 426-year drought history for Western Tian Shan, Central Asia, inferred from tree-rings and its linkages to the North Atlantic and Indo–West Pacific Oceans. Holocene.

[39] Chen F, Yu S (2017). Tree-ring indicators of rainfall and streamflow for the Ili-Balkhash Basin, Central Asia since CE 1560. Paleogeo, Paleoclim, Paleocolo.

[40] Chen F (2015). Climatic signals in tree rings of Juniperus turkestanica in the Gulcha River Basin (Kyrgyzstan) reveals the recent wetting trend of highAsia. Dendrobiology.

[41] Zhang RB (2017). Tree-ring-based precipitation reconstruction in southern Kazakhstan, reveals drought variability since A.D. 1770. Inter. J. Clima..

[42] Burn DH, Hag EMA (2002). Detection of hydrological trends and variability. J. Hydro..

